# Vascularized jejunal pedicle graft for duodenal reconstruction in a cat: Case Report

**DOI:** 10.3389/fvets.2026.1760217

**Published:** 2026-04-17

**Authors:** A. Jones, G. Hayes

**Affiliations:** Department of Clinical Studies, College of Veterinary Medicine, Cornell University, Ithaca, NY, United States

**Keywords:** cat, duodenal reconstruction, feline, flap, pedicle, vascular

## Abstract

Reconstruction of large defects of the proximal duodenum can be challenging, as sacrifice of this region requires biliary re-routing ± partial pancreatectomy, resulting in considerable morbidity. This article reports a novel method for duodenal reconstruction with long-term follow-up. The technique was performed after resection of a large enteric duplication cyst causing intestinal obstruction in a 1-year-old spayed female domestic short hair cat presented for vomiting. A 2.7 × 2.3 cm hemi-circumferential cyst involving the left duodenal wall, located adjacent and caudal to the major duodenal papilla, was identified. Following full-thickness resection with preservation of the pancreatic-duodenal artery and vein, the resulting defect was reconstructed with a vascularized jejunal pedicle graft harvested from the mid-jejunum. The donor site was closed with an end-to-end anastomosis. The pedicle graft retained complete viability, with no evidence of stricture or stenosis of the recipient region on follow-up imaging at 9 months post-operatively. This method may be considered for the effective repair of large mural defects in the duodenum.

## Introduction

Repair and reconstruction of large proximal duodenal defects may be difficult due to the presence of the major duodenal papilla opening in this area, as well as a shared blood supply with the right limb of the pancreas (1). Large duodenal wall defects are often converted to a circumferential transection due to the inability to achieve primary repair without substantial stenosis at the repair site. Circumferential duodenal resection often requires extensive pancreatic dissection or a concurrent partial pancreatectomy of the right limb of the pancreas due to the shared blood supply between the pancreas and the duodenal wall through the pancreaticoduodenal artery and vein (1, 2). Circumferential resections that include the duodenal papilla or the intramural segment of the common bile duct also require either common bile duct re-implantation or biliary re-routing (3, 4). As the primary source of motilin, extensive resection of the duodenum can result in gastroparesis with associated chronic clinical signs (5). While the morbidity that follows all of these interventions can be managed, a surgical option that avoids circumferential resection while maintaining normal lumen diameter would appear desirable.

This case report describes the use of a vascularized jejunal pedicle graft to reconstruct a large hemi-circumferential defect in the proximal duodenal wall adjacent to the duodenal papilla in a cat. Repeat imaging performed 1-year post-repair showed full incorporation of the graft with no evidence of stenosis at the graft site.

## Case presentation

A 1-year-old female spayed domestic short-hair cat weighing 3.5 kg was referred to the Cornell University Hospital for Animals for a 2-week history of intermittent bilious vomiting, anorexia, and excessive grooming behavior. Survey abdominal radiographs performed by the referring veterinarian revealed a mid-abdominal mass effect without evidence of segmental enteropathy or loss of serosal detail. On presentation, the cat was quiet, alert, and responsive with a mildly elevated rectal temperature (39.8 °C), tachycardia (260 beats/min), and tachypnea (56 breaths/min) with normal respiratory effort. Physical examination identified 5–7% dehydration and revealed multifocal alopecia regions on the ventral abdomen and distal limbs, together with a tense, non-painful abdomen with a firm palpable mass in the right cranial quadrant. The remainder of the physical examination was unremarkable. Venous blood gas analysis showed an unremarkable acid–base and electrolyte status with mild hyperlactatemia (2.73 mmol/L, reference range, <2.0 mmol/L). The cat was hospitalized for correction of dehydration and further diagnostic evaluation of the abdominal mass. Initial stabilization included peripheral intravenous catheter placement and crystalloid fluid administration consisting of an initial IV bolus of 15 mL/kg plasmalyte followed by a rate of 90 mL/kg/day, along with maropitant (1 mg/kg IV, every 24 h), pantoprazole (1 mg/kg IV, every 12 h), ondansetron (0.5 mg/kg IV, every 8 h), gabapentin (14 mg/kg PO, every 8 h), and methadone (0.2 mg/kg IV, every 6 h as needed).

A complete blood count and electrolyte profile performed following crystalloid fluid therapy were within normal limits. Abnormal serum biochemical findings included mild aspartate aminotransferase elevation (76 U/L, reference range: 17–48 U/L), moderate creatinine kinase elevation (2,619 U/L, reference range: 74–386 U/L), and mildly elevated serum lipase (30 U/L, reference range: 7–23 U/L). The retroviral testing (feline immunodeficiency virus and feline leukemia virus) and the heartworm antigen testing were negative.

### Imaging

Abdominal ultrasonography revealed a 2.8 cm diameter mass arising from the dorsal wall of the descending duodenum immediately aborad to the duodenal papilla. The lesion displayed organized wall layering and two connected internal cavities containing anechoic fluid, without evidence of overt communication with the duodenal lumen. Scant anechoic peritoneal effusion was detected; diagnostic abdominocentesis was not performed due to the minimal volume and diffuse distribution of the effusion. A small volume of echogenic, gravity-dependent gallbladder sediment was noted, with no evidence of intrahepatic or extrahepatic biliary duct dilation. The remainder of the abdominal ultrasound evaluation was unremarkable.

**Figure 1:**
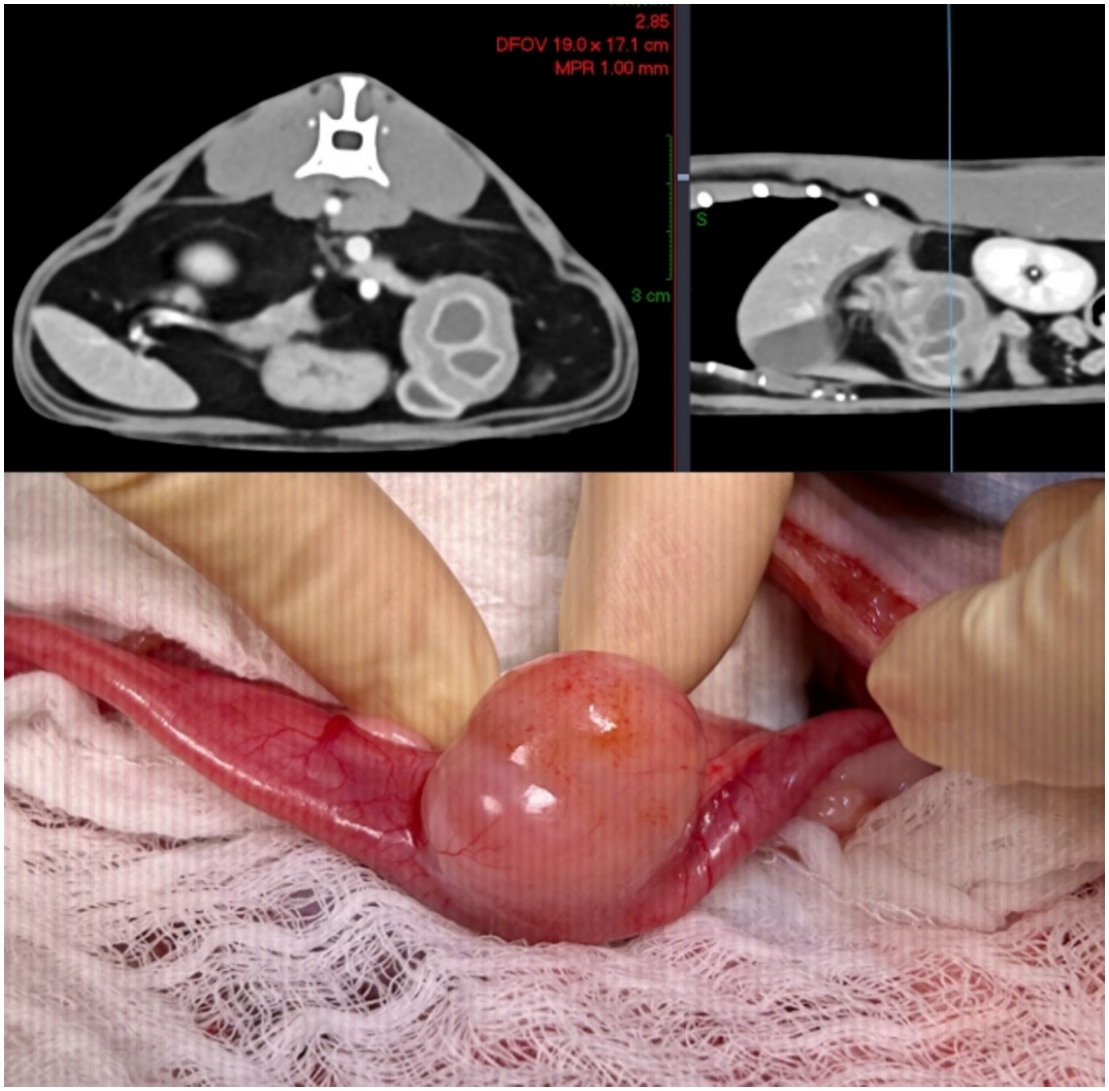
Enteric duplication cyst on computed tomographic imaging and intra-operatively, showing duodenal luminal compression.

Computed tomography of the thorax and abdomen was then performed for further characterization of the identified duodenal pathology. Pre- and post-contrast images were acquired in 1 mm contiguous transverse slices following administration of iohexol contrast medium (740 mg/kg IV) and reconstructed into 0.8-mm multiplanar volumes. A 2.7 cm × 2.3 cm mass arising from the dorsal duodenal wall and abutting the right pancreatic limb was identified, containing two internal cavities containing fluid-attenuating medium (HU 37–40) and secondary mild ventral compression of the orad descending duodenum (Figure 1). The previously noted scant volume of peritoneal effusion and gallbladder sediment was not detected. Additional findings of unknown clinical significance included two small (0.5 cm diameter) fat-attenuating nodules dorsal to the gastric fundus and multifocal mild lymphadenopathy of the thorax and abdomen. Computed tomography (CT) detected a large, solitary presumed duodenal duplication cyst with suspected dynamic mechanical obstruction of the duodenum, prompting a recommendation for exploratory laparotomy and resection.

### Surgical technique

The cat was premedicated for general anesthesia with methadone (0.2 mg/kg IV) and dexmedetomidine (0.7 μg/kg IV). General anesthesia was induced using alfaxalone (5 mg/kg IV, to effect) and maintained with isoflurane in 100% oxygen. The cat was administered with plasmalyte infusion at a constant rate of 2.5 mL/kg/h, followed by the administration of ampicillin–sulbactam (30 mg/kg IV) prophylactically pre-operatively, and every 90 min thereafter intra-operatively. Intra-operative hypotension was responsive to administration of ephedrine (0.1 mg/kg IV, single dose) and norepinephrine (0.05–0.25 μg/kg/min) as a constant rate infusion titrated to effect.

The patient was positioned in dorsal recumbency, and the ventral abdomen was clipped, prepped, and draped in a standard aseptic fashion. A routine ventral midline laparotomy was performed, and exploration revealed a firm, bilobed, subserosal mass in the proximal duodenum extending from the antimesenteric to mesenteric border at the level of the right limb of the pancreas (Figure 1). The remainder of the abdomen was unremarkable. Initial dissection of the lesion confirmed that the mass formed an integral part of the duodenal wall. The adjacent regions of the duodenum were digitally occluded, and the mass was excised en bloc with a 1 mm margin. A 2 mm region of cystic wall was retained immediately adjacent to the right limb of the pancreas at the level of the mesoduodenum to allow preservation of the pancreaticoduodenal artery. Following excision, a defect approximately 3.5 cm x 2 cm was present in the duodenal wall. Location and patency of the major duodenal papilla, approximately 1 cm orad to the defect, were confirmed during excision through the presence of biliary flow from the orifice upon gallbladder expression.

**Figure 2:**
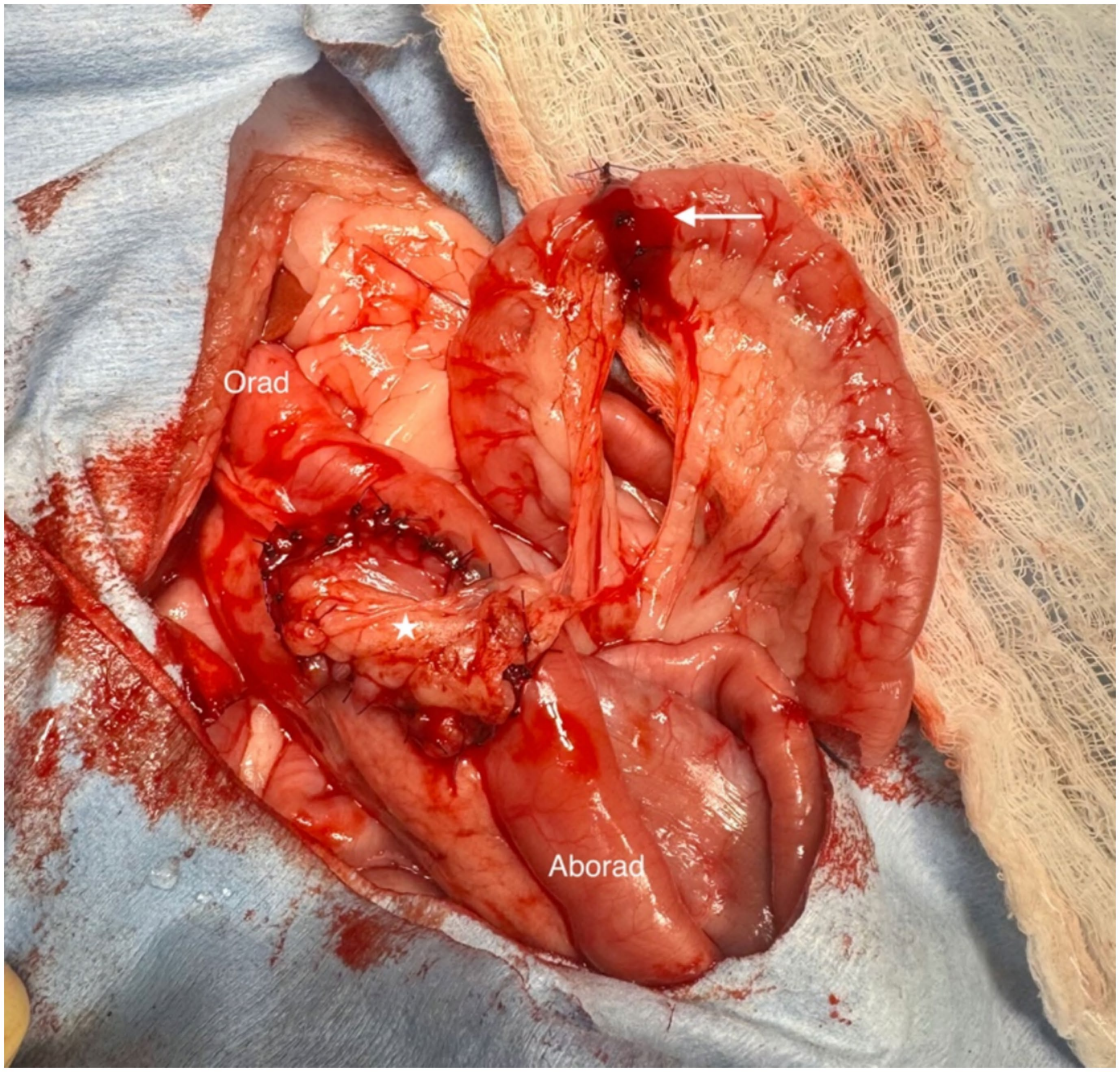
Vascularized jejunal pedicle graft. White arrow; donor site. White star; recipient site.

An approximately 4-cm-long segment of jejunum was identified as the donor site for harvesting the vascularized pedicle graft. Identification of donor location was based upon anticipated tension applied to the vascular pedicle due to the distance to the recipient site and the length of the native vascular supply at the proposed donor location. The mesojejunum on either side of the identified donor site was sharply incised, extending toward the root of the mesentery. The perimural mesenteric artery and vein were double ligated at each end of the proposed donor site using 4-0 polydioxanone (4-0 PDS) and transected between the two ligatures. The jejunum on either side of the anticipated donor site was occluded by application of atraumatic intestinal forceps, and the margins of the donor segment were sharply transected. The donor segment was then incised at the antimesenteric border, and the resulting rectangular, vascularized patch was trimmed to the size of the duodenal defect, creating an oval patch with an associated vascular pedicle. The segment was transposed and applied to the duodenal defect. The jejunal vascularized pedicle graft was then sutured to the duodenal recipient site using 4-0 PDS in a simple interrupted pattern (Figure 2). Simultaneously, an end-to-end anastomosis of the jejunal donor site was performed using 4-0 PDS in a simple interrupted pattern. Integrity of the pedicle graft site and donor anastomosis site was assessed by intra-luminal injection of approximately 15 mL sterile saline, following digital occlusion, with no leakage observed. The abdominal cavity was copiously lavaged with warm sterile saline, and the anastomosis sites were wrapped with greater omentum prior to routine closure of the linea alba using 2-0 PDS in a simple continuous pattern. The subcutaneous tissue was closed using 2-0 poliglecaprone 25, followed by an intradermal pattern using 3–0 poliglecaprone 25.

The total surgical time was 134 min. Recovery from general anesthesia was uneventful and the cat was hospitalized for post-operative management comprised of plasmalyte infusion at a constant rate of 60 mL/kg/day, maropitant (1 mg/kg IV, every 24 h), pantoprazole (1 mg/kg IV, every 12 h), gabapentin (14 mg/kg PO, every 8 h), ampicillin–sulbactam (30 mg/kg IV, every 8 h), and buprenorphine (0.02 mg/kg IV, every 8 h as needed). The cat demonstrated a return to appetite and resolution of vomiting during the immediate post-operative period and was discharged 24 h post-operatively with instructions for activity restriction, incision care, and systemic monitoring. Outpatient management comprised gabapentin (14 mg/kg PO, every 8 h as needed), buprenorphine (0.015 mg/kg PO, every 8 h as needed), and omeprazole (1 mg/kg PO, every 12 h).

### Outcome

Histologic evaluation of the excised duodenal lesion was consistent with a clinically suspected enteric duplication cyst. The cystic structure expanded tunica muscularis and serosa and was delineated by an irregular, multilayered smooth muscle wall that blended with the tunica muscularis of the duodenum. The cystic lining was composed of a simple columnar epithelium with occasional intestinal crypt-like structures. The adjacent native duodenal mucosa was infiltrated by moderate numbers of plasma cells, lymphocytes, and neutrophils, consistent with mild lymphoplasmacytic duodenitis, presumed secondary to the concurrent enteric duplication cyst; however, histologic manifestation of a concurrent underlying enteropathy could not be excluded.

### Follow-up

**Figure 3:**
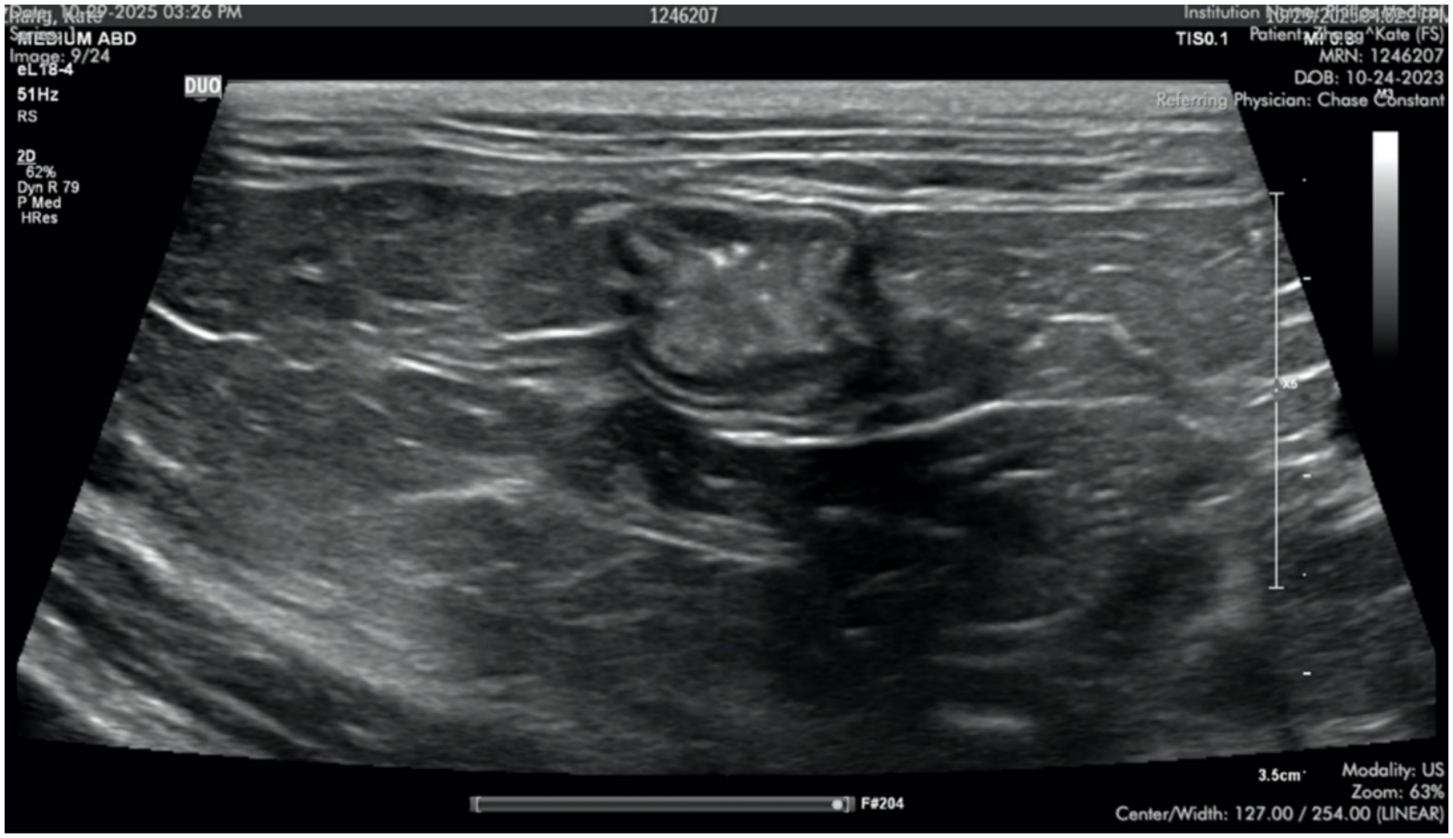
Outpouching of the duodenum at the graft site, 9-month post-surgery, on abdominal ultrasound.

Nine months following the surgical procedure, the cat was reassessed. Appetite was normal, no vomiting had been observed, and his body weight had increased by 0.4 kg. CBC and chemistry profiles revealed no abnormalities. Abdominal ultrasound showed a normal biliary tree. Immediately aborad to the duodenal papilla, there was a focal mild outpouching or widening of the duodenum for a short distance before transitioning back to normal diameter (Figure 3). The duodenal wall showed normal layers and thickness at this site. There was no evidence of stricture or recurrence of the duplication cyst.

## Discussion

Enteric duplication cysts are uncommon malformations that can form during embryonic development at any level of the gastrointestinal tract (6). Diagnosis of an enteric duplication cyst requires the histological identification of a smooth muscle layer and an alimentary epithelial lining, which may arise from the adjacent bowel or from an ectopic site (7). The precise etiology of enteric duplication cysts is unclear; however, the suggested pathophysiology is speculated to include failure of normal canalization of the gastrointestinal tract or formation of abnormal connections between the neural ectoderm and intestinal endoderm secondary to intrauterine mechanical traction or ischemic infarction during development (7, 8). The duplication cyst may be directly communicating with the adjacent lumen or contiguous but non-communicating, and exhibit a variable degree of clinical manifestation, ranging from incidental findings to complete mechanical obstruction caused by luminal compression or intussusception (8). Enteric duplication cysts have been described in dogs, cats, horses, and goats, and are reported most commonly in the pediatric population in the human literature (6, 9). The ileum and esophagus are the most frequently reported location in the human literature; however, in cats, these cysts are most commonly reported in the duodenum, including a documented incidence of multiple level duplication cysts by Bernarde et al. (9, 10) While most cysts represent benign congenital anomaly, rare malignant transformation of the cysts has been reported in humans and a single cat in the veterinary literature by Hobbs et al., in which the cyst was histologically consistent with duodenal adenocarcinoma (11). Acute severe pancreatitis due to obstruction of the duodenal papillae and life-threatening hemorrhage or ulceration at the site of duplication as a result of adjacent or ectopic gastric mucosa have also been reported as uncommon sequelae (6, 12, 13).

Open surgical intervention is the most common therapeutic approach for enteric duplication cysts in both the human and veterinary literature. Complete surgical resection of the duplication is frequently feasible and is recommended to prevent recurrence of clinical signs or rare malignant transformation. Small intestinal duplication cysts have been documented to affect either the antimesenteric or mesenteric border in cats, which is in contrast to the typically mesenteric orientation in humans (7, 14). The present case report is an example of a non-communicative duplication cyst with extension from the antimesenteric to mesenteric border of the proximal duodenum. The clinical decision to perform a vascularized jejunal pedicle graft in this case was based on several considerations. Debulking of the non-communicative portion of the enteric duplication cyst, followed by omentalization, as previously described, was not chosen due to concern for the risk of persistent dynamic mechanical obstruction (9). The orientation and location of duplication immediately aborad to the major duodenal papilla limited routine surgical resection and anastamosis without concurrent re-routing of the biliary tree and partial pancreatectomy. In the human literature, the treatment choice for duplication cysts in this region that encroach upon the pancreatic head and biliary tract is the performance of a pancreaticoduodenectomy unless an identified candidate for endoscopic marsupialization (6). However, considering the benign character of the clinically suspected duplication cyst and the morbidity associated with pancreaticoduodenectomy and biliary re-routing, an alternative marginal resection was elected in the present case. Due to the length of the lesion and expansion across approximately 50% of the luminal circumference, primary closure was not considered feasible without severe duodenal stenosis. Primary closure with duodenal stenosis and concurrent gastrojejunostomy to bypass the stenosis was considered, with the goal of maintaining native patency of the biliary and pancreatic ducts through the stenotic area while allowing food ingesta to bypass the area. Gastrojejunostomy has been used infrequently in this context in the veterinary literature, most commonly for palliative management of stricture and inoperable or metastatic lesions of the upper gastrointestinal tract; however, this anastomosis is associated with post-operative morbidity, including chronic gastrointestinal signs and weight loss due to rapid gastric emptying, and risk of anastomotic ulceration (15). As an alternative, this study utilized a vascularized pedicle jejunal graft to resolve mechanical obstruction, maintain adequate luminal diameter, and limit post-operative morbidity by eliminating the need for biliary rerouting or partial pancreatectomy.

Numerous small intestinal grafting techniques, including pedicled, augmented, free segment, or combination approaches, have been utilized for reconstructing anatomical defects of varying anatomic sites in the review of the human patient literature (16–18). The surgical technique of duodenal reconstruction through jejunal pedicle graft was described in detail by Bensignor et al., and its superior histologic characteristics were supported by an experimental animal-based model in comparison of pedicled and serosal patching in dogs (19, 20). The use of vascularized small intestinal pedicle grafts has also emerged in the recent clinical veterinary literature. Case reports of jejunal pedicle grafting for management of duodenal and colorectal defects, together with additional distant anatomic sites, have been reported with successful clinical outcomes in dogs (16–18). Novel literature supporting the clinical use of small intestinal pedicle grafting is limited in cats, with the exception of ileal grafting for management of ureteral obstruction by Brourman et al. (21). To the authors’ knowledge, this is the first clinical report of pedicled jejunal grafting in a cat for reconstructing a large duodenal defect.

The jejunum is considered an ideal grafting site for the restoration of duodenal continuity. The jejunum permits standard resection and anastomosis with minimal long-term morbidity upon recovery in the absence of complications. Modification of the length of donor site resection is permitted, dependent upon recipient defect size and the character of the mesojejunum and associated vasculature, which permits adequate segment mobility for grafting without excessive tension (16). Jejunal grafts have additionally been confirmed to demonstrate peristaltic function in canine models, suggesting retained baseline motility (22, 23). The present case recovered without immediate or long-term surgical complications; however, possible reported complications in the use of jejunal grafts include post-operative stenosis, vascular thrombosis, or vascular obstruction secondary to tension or irregular angulation (16, 18). Dysmotility has also been rarely reported, likely secondary to poor coordination in functional motility at the site of grafting (16). Utilization of a jejunal pedicle graft also necessitates performance of an anastomosis at the donor site, inherently increasing surgical time and the potential for complications, including dehiscence and stricture. However, with mindful selection of a grossly normal, well-perfused donor site and absence of known risk factors for dehiscence, the anticipated risk of jejunojejunal anastomosis dehiscence is minimal in the cat (24).

The outcome of this case supports consideration of jejunal pedicle grafting in cases of benign duodenal pathology without the need for local oncologic disease control in cats. This technique permits the maintenance of an appropriate luminal diameter and avoids the morbidity associated with upper gastrointestinal re-routing procedures. Larger studies are indicated to further characterize the long-term clinical outcomes and complication profile of vascularized jejunal pedicle graft use in cats.

## Data Availability

The original contributions presented in the study are included in the article/supplementary material, further inquiries can be directed to the corresponding author.
